# Rationale and design of repeated cross-sectional studies to evaluate the reporting quality of trial protocols: the Adherence to SPIrit REcommendations (ASPIRE) study and associated projects

**DOI:** 10.1186/s13063-020-04808-y

**Published:** 2020-10-28

**Authors:** Dmitry Gryaznov, Ayodele Odutayo, Belinda von Niederhäusern, Benjamin Speich, Benjamin Kasenda, Elena Ojeda-Ruiz, Anette Blümle, Stefan Schandelmaier, Dominik Mertz, Yuki Tomonaga, Alain Amstutz, Christiane Pauli-Magnus, Viktoria Gloy, Karin Bischoff, Katharina Wollmann, Laura Rehner, Szimonetta Lohner, Joerg J. Meerpohl, Alain Nordmann, Katharina Klatte, Nilabh Ghosh, Ala Taji Heravi, Jacqueline Wong, Ngai Chow, Patrick Jiho Hong, Kimberly Mc Cord, Sirintip Sricharoenchai, Jason W. Busse, Arnav Agarwal, Ramon Saccilotto, Matthias Schwenkglenks, Giusi Moffa, Lars G. Hemkens, Sally Hopewell, Erik von Elm, Matthias Briel

**Affiliations:** 1 Department of Clinical Research, Basel Institute for Clinical Epidemiology and Biostatistics, University Hospital Basel and University of Basel, Spitalstrasse 12, 4031 Basel, Switzerland; 2 Applied Health Research Centre, Li Ka Shing Knowledge Instiute of St Michael’s Hospital, Toronto, Canada; 3 Oxford Clinical Trials Research Unit and Centre for Statistics in Medicine, Nuffield Department of Orthopaedics, Rheumatology and Musculoskeletal Sciences, University of Oxford, Oxford, UK; 4 Department of Clinical Research, Clinical Trial Unit, University Hospital Basel and University of Basel, Basel, Switzerland; 5 Roche Pharma AG, Grenzach-Wyhlen, Germany; 6 Department of Medical Oncology, University Hospital Basel, Basel, Switzerland; 7 iOMEDICO AG, Research & Development, Freiburg, Germany; 8 Preventive Medicine Department, Osakidetza Basque Health Service, Bioaraba Health Research Institute, Health Prevention, Promotion and Care Area, Araba University Hospital, Vitoria-Gasteiz, Spain; 9 Institute for Evidence in Medicine, Medical Center – University of Freiburg, Faculty of Medicine, University of Freiburg, Freiburg, Germany; 10 Cochrane Germany, Cochrane Germany Foundation, Freiburg, Germany; 11 Department of Health Research Methods, Evidence, and Impact, McMaster University, Hamilton, Canada; 12 Epidemiology, Biostatistics and Prevention Institute, University of Zurich, Zurich, Switzerland; 13 Swiss Tropical and Public Health Institute, University of Basel, Basel, Switzerland; 14 Department of Infectious Diseases and Hospital Epidemiology, University Hospital Basel, Basel, Switzerland; 15 Department of Epidemiology and Community Health, Institute for Community Medicine, University Medicine Greifswald, Greifswald, Germany; 16 Cochrane Hungary, Clinical Centre of the University of Pécs, Medical School, University of Pécs, Pécs, Hungary; 17 Department of Neurosurgery and Department of Biomedicine, University Hospital Basel, University of Basel, Basel, Switzerland; 18 Department of Anesthesiology and Pain Medicine, University of Toronto, Toronto, Canada; 19 Department of Anesthesia, McMaster University, Hamilton, Canada; 20 Institute of Pharmaceutical Medicine (ECPM), University of Basel, Basel, Switzerland; 21 Department of Mathematics and Computer Science, University of Basel, Basel, Switzerland; 22 Cochrane Switzerland, Centre for Primary Care and Public Health (Unisanté), University of Lausanne, Lausanne, Switzerland

**Keywords:** Randomized clinical trials, Trial protocol, Reporting quality, Reporting guideline adherence, Registration, Trial discontinuation

## Abstract

**Background:**

Clearly structured and comprehensive protocols are an essential component to ensure safety of participants, data validity, successful conduct, and credibility of results of randomized clinical trials (RCTs). Funding agencies, research ethics committees (RECs), regulatory agencies, medical journals, systematic reviewers, and other stakeholders rely on protocols to appraise the conduct and reporting of RCTs. In response to evidence of poor protocol quality, the Standard Protocol Items: Recommendations for Interventional Trials (SPIRIT) guideline was published in 2013 to improve the accuracy and completeness of clinical trial protocols. The impact of these recommendations on protocol completeness and associations between protocol completeness and successful RCT conduct and publication remain uncertain.

**Objectives and methods:**

Aims of the Adherence to SPIrit REcommendations (ASPIRE) study are to investigate adherence to SPIRIT checklist items of RCT protocols approved by RECs in the UK, Switzerland, Germany, and Canada before (2012) and after (2016) the publication of the SPIRIT guidelines; determine protocol features associated with non-adherence to SPIRIT checklist items; and assess potential differences in adherence across countries.

We assembled an international cohort of RCTs based on 450 protocols approved in 2012 and 402 protocols approved in 2016 by RECs in Switzerland, the UK, Germany, and Canada. We will extract data on RCT characteristics and adherence to SPIRIT for all included protocols. We will use multivariable regression models to investigate temporal changes in SPIRIT adherence, differences across countries, and associations between SPIRIT adherence of protocols with RCT registration, completion, and publication of results.

We plan substudies to examine the registration, premature discontinuation, and non-publication of RCTs; the use of patient-reported outcomes in RCT protocols; SPIRIT adherence of RCT protocols with non-regulated interventions; the planning of RCT subgroup analyses; and the use of routinely collected data for RCTs.

**Discussion:**

The ASPIRE study and associated substudies will provide important information on the impact of measures to improve the reporting of RCT protocols and on multiple aspects of RCT design, trial registration, premature discontinuation, and non-publication of RCTs observing potential changes over time.

**Supplementary information:**

**Supplementary information** accompanies this paper at 10.1186/s13063-020-04808-y.

## Introduction

Protocols are essential documents for the planning, conduct, and reporting of randomized clinical trials (RCTs) [1]. Empirical studies investigating cohorts of clinical trial protocols from the 1990s found the reporting quality of RCT protocols to be limited, specifically in the description of treatment allocation methods, primary outcomes, use of blinding, adverse events reporting, sample size calculations, data analysis, and the roles of sponsors in trial design or access to data [2–9].

### Ethical and practical implications of deficient trial protocols

Inadequately reported or incomplete RCT protocols may have serious implications for sponsors, trial staff, involved patients, systematic reviewers, and other users of trial results. A lack of key elements in a protocol may lead to missing or unreliable data compromising the validity of trial results; low quality trial protocols may be associated with insufficient planning and unsuccessful conduct of a trial, premature trial discontinuation, and eventual non-publication [10]—potentially putting participants at unnecessary risk with minimal scientific return on the investment [11, 12]. In addition, participants may suffer due to ill-informed treatment decisions based on compromised trial evidence. If important details are missing from the protocol, peer reviewers, journal editors, clinicians, or systematic reviewers may not be able to identify discrepancies between the published report of a clinical trial and the protocol [4], which can be relevant, for instance, when judging the credibility of subgroup effects or identifying measured but not reported outcomes [4, 13, 14].

### SPIRIT—reporting guideline for trial protocols

In 2007, the Standard Protocol Items: Recommendations for Interventional Trials (SPIRIT) Initiative began working to improve the completeness of clinical trial protocols and, in January 2013, published evidence-based recommendations for a minimum set of items to be addressed in trial protocols [12, 15]. According to the Web of Science, the SPIRIT publications have been cited almost 3000 times, as of September 25, 2020. An important aspect of the implementation of the SPIRIT guideline is to evaluate its impact on the reporting quality of trial protocols over time. So far, there are only a few studies that have used [16] or plan to use [17] the SPIRIT checklist as a tool to assess the completeness of trial protocols. Kyte et al. investigated patient-reported outcomes in 75 RCT protocols supported by the UK National Institute for Health Research (NIHR) Health Technology Assessment (HTA) programme from 2012 and 2013 and examined whether the quality of reporting of patient-reported outcomes was associated with overall protocol completeness [16]. They found that protocols adhered on average to 63% of 51 SPIRIT recommendations giving items and subitems of the checklist equal weights. Madden et al. focused in a planned study on the reporting of the statistics section in published surgical trial protocols using 11 items from the SPIRIT checklist [17]. Thabane et al. plan to assess the reporting quality of cluster randomized trials with a stepped wedge design, including protocols of such trials using the SPIRIT checklist, but they do not provide details about which SPIRIT items or subitems will be assessed and how these will be weighted in their analysis [18].

### Rationale for meta-epidemiological research with RCT protocols

Clearly structured and comprehensive study protocols are essential to ensure the safety and well-being of study participants, data validity, and credibility of results, particularly in the case of RCTs. Incomplete protocols jeopardize all stages of the clinical research process with potentially harmful consequences for patients, decision-making in health care, the scientific community, and society as a whole. Since most evidence on the accuracy and completeness of trial protocols dates back to the 1990s, empirical evidence from more recent protocols is needed. In particular, the potential effect of the publication of the SPIRIT recommendations on the quality of RCT protocols remains unclear.

The Adherence to SPIrit REcommendations (ASPIRE) study group is an international collaboration of researchers which aims to evaluate the completeness of RCT protocols approved by RECs in the UK (Bristol regional office), Switzerland (Basel, Bern, Geneva, Lausanne, St. Gallen, Thurgau [from 2016 together with St. Gallen], Bellinzona, and Zurich), Germany (Freiburg), and Canada (Hamilton) before publication of the SPIRIT statement (in 2012) and thereafter (in 2016).

In addition to recently published work on phase I trials and multi-arm trials [19, 20], the secondary objectives of the ASPIRE study include examining trial registration, premature discontinuation, and non-publication of RCTs; the use of patient-reported outcomes and specifically of health-related quality of life (HRQoL) outcomes in RCT protocols; the protocol quality of RCTs with regulated vs non-regulated interventions; the planning of subgroup analyses in RCT protocols; and the planned use of routinely collected data in RCTs.

## Study objectives

Our focus is on RCTs because their results typically impact clinical practice and guidelines. Furthermore, participants in RCTs are exposed to risks and burdens that invoke a fiduciary responsibility on the part of clinical trial investigators to ensure that the trial is conducted to the highest methodological standard.

### Specific objectives of the ASPIRE study are as follows:

1. To compare the completeness of RCT protocols approved by RECs in the UK, Switzerland, Germany, and Canada before (2012) the publication of the SPIRIT checklist (January 2013) and thereafter (2016) based on the protocol adherence to SPIRIT checklist items.
2. To determine trial characteristics associated with non-adherence to SPIRIT checklist items including potential interactions between year of approval (2012 or 2016) and sponsorship of protocols, and year of approval (2012 or 2016) and reported methodological support from Clinical Trial Units or Clinical Research Organisations.
3. To investigate whether the comprehensiveness of RCT protocols is different across countries (Switzerland, Germany, Canada, the UK).

### Additional objectives and rationales for substudies of ASPIRE

1. Subproject 1, DISCOntinued trials (DISCO) II: Our previous study of RCT protocols approved by Swiss, Canadian, or German RECs between 2000 and 2003 found that one out of four initiated RCTs was prematurely discontinued and that only 70% of completed and less than 50% of discontinued RCTs were published in peer-reviewed journals [10]. This is especially worrying, as results from published trials are systematically different from unpublished trials [21–23]. Public trial registries are meant to provide a comprehensive overview of all ongoing clinical trials, which can help reduce duplication in research and minimize publication bias [24]. However, even though the International Committee of Medical Journal Editors (ICMJE) mandated prospective registration of trials which started enrolment after July 2005 as a requirement for publication, it is still common for RCTs to be registered after completion or not at all [25, 26]. Furthermore, there are often discrepancies between data in trial registries and the corresponding publication of an RCT with respect to important items, such as the primary outcome [26].

We will use RCTs included in the ASPIRE study (the UK, Switzerland, Germany, and Canada) to evaluate: (i) the extent of registered and, in particular, prospectively registered protocols in national or international registries; (ii) the proportion of prematurely discontinued RCTs and reasons for discontinuation; (iii) the proportion of RCTs not published in a peer-reviewed journal or without results posted in a public trial registry; (iv) the extent to which unpublished RCTs (in particular those that were prematurely discontinued) can be identified through trial registries; (v) whether the proportions of trials under categories (i)–(iv) vary across RECs in different countries; and (vi) whether the completeness of reporting of RCT protocols according to SPIRIT is associated with the proportion of discontinued RCTs due to poor recruitment or non-publication of RCT results (i.e. neither in a peer-reviewed journal, nor in a trial registry). We propose to compare the RCT cohorts from 2012 and 2016 with RCTs approved 2000–2003 from the previous DISCO study [10, 27]. We will also investigate the agreement of trial characteristics between the approved study protocols (later amendments considered), registry data, and journal publications (e.g. primary outcome, patient eligibility criteria).

2. Subproject 2, Patient-reported outcomes: Patient-reported outcomes and specifically the subgroup of HRQoL outcomes are highly relevant for decision making in health care and policy [28–30]. Nonetheless, patient-reported outcomes, including HRQoL, are infrequently considered in RCTs [31], or specified in protocols but not reported in RCT publications [31–34].
  We plan to investigate the prevalence and characteristics of patient-reported outcomes in RCT protocols approved in 2012 and 2016 and their reporting in corresponding publications. In particular, we will compare the prevalence of HRQoL outcomes in protocol cohorts of 2012 and 2016 with RCT protocols approved in 2000–2003 [34].
3. Subproject 3, Non-regulated interventions: Trials with “regulated interventions” such as drugs, biologics, or medical devices are controlled by regulatory agencies; trials with “non-regulated interventions” such as dietary interventions, surgical procedures, behavioural and lifestyle interventions, or exercise programmes are not reviewed by regulatory agencies. RCTs evaluating regulated interventions may, therefore, be associated with higher quality protocols, greater likelihood of registration, lower risk for selective outcome reporting bias, and a higher likelihood of publication [35–39].
  We will determine the prevalence of RCTs evaluating non-regulated interventions and investigate whether their associated protocols are associated with lower adherence to SPIRIT recommendations in comparison with RCT protocols testing regulated interventions. In addition, we will identify individual SPIRIT checklist items with lowest adherence in RCT protocols testing non-regulated interventions.
4. Subproject 4, Subgroups: In a previous study with RCT protocols approved between 2000 and 2003, we found that almost 30% of protocols included one or more planned subgroup analyses [13]; however, most were poorly reported: Only 7% provided a clear hypothesis for at least one subgroup analysis, 4% anticipated the direction of a subgroup effect, and 35% planned a statistical test for interaction. Industry-sponsored trials more often planned subgroup analyses compared with investigator-sponsored trials (35% versus 17%).
  We will investigate if the prevalence and description of subgroup analyses in RCT protocols from 2012 and 2016, stratified by medical discipline, differ from those approved in the early 2000s [13]. In addition, we will assess the percentage of planned subgroup analyses based on molecular markers in RCT protocols from 2012 and 2016.
5. Subproject 5, Routinely collected data: Using routinely collected data may facilitate the planning and conduct of RCTs [40]. For instance, data from electronic health records (EHRs), registries, or administrative claims data can be used to efficiently collect outcome data for RCTs, or targeted screening of routine data may enhance the recruitment of eligible patients. It is unclear how often and for which purposes routinely collected data are actually used in RCTs.
  We will investigate the prevalence, characteristics, and purposes of routinely collected data for RCTs described in protocols from 2012 and 2016, stratified by medical discipline.

## Methods

This meta-research study will be conducted by the Adherence to SPIrit REcommendations (ASPIRE) study group, an international collaborative group of researchers involving all RECs in Switzerland (Basel, Bellinzona, Bern, Geneva, Lausanne, St. Gallen, Thurgau (from 2016 together with St. Gallen), Zurich), as well as one REC in Germany (Freiburg), one REC in Canada (Hamilton), and the Bristol office of the UK National Research Ethics Service (which is responsible for 19 RECs in the UK). We have obtained support and established cooperation with the aforementioned RECs, building on successfully completed prior research [10].

While the main ASPIRE study examining SPIRIT checklist items was conceived as a joint analysis among all involved RECs, there were differences in the timeline to access protocols from 2016 in different countries. As such, the main ASPIRE study will be conducted separately for the UK RECs.

### Eligibility criteria for study sample

We will include protocols of all approved RCTs in 2012 and 2016 that compared an intervention with placebo, a sham intervention, another active intervention, or no intervention or combinations thereof. We define an RCT as a prospective study in which patients, or groups of patients, are assigned at random to one or more interventions to evaluate their effect on health outcomes. Studies comparing different doses or routes of administration of the same drug and trials labelled as pilot or feasibility studies will be included but represent two pre-specified subgroups. We will exclude studies enrolling healthy volunteers (e.g. pharmacokinetic studies, training interventions in sport science), economic evaluations, animal studies, studies based on tissue samples, observational studies, studies involving only qualitative methods, and studies with a quasi-random method of allocation.

### RCT selection process

**Fig. 1:**
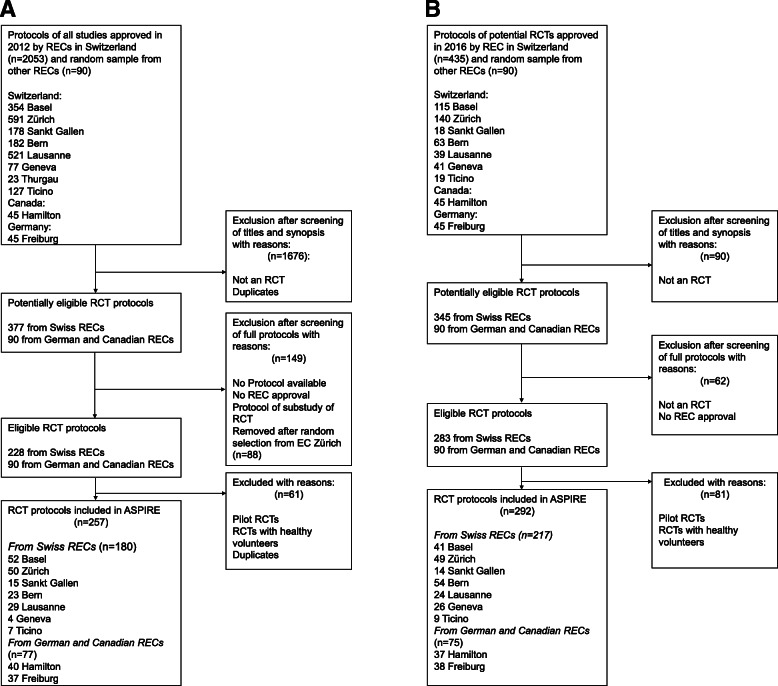
**a** Flow diagram for included randomized clinical trial protocols in ASPIRE with ethics approval in 2012. **b** Flow diagram for included randomized clinical trial protocols in ASPIRE with ethics approval in 2016

We have already screened in detail all studies approved by RECs in Switzerland, in Freiburg (Germany), and in Hamilton (Canada) in 2012 and 2016 using the above-described criteria. For feasibility reasons, we have acquired a stratified (by tertile of submission) random sample of 45 studies per year from protocols approved in Freiburg and Hamilton. In addition, we drew a stratified random sample of 60 protocols out of all 148 eligible RCT protocols approved by the REC in Zurich in 2012. Figure 1a and b illustrate the RCT selection process for RECs in Switzerland, Germany, and Canada. Detailed flow diagrams illustrating the selection process for protocols from RECs in the UK will be provided at a later stage.

### Confidentiality when handling RCT protocols

The involved Swiss, German, UK, and Canadian RECs are all project partners, and we are collaborating with a mandate from each participating REC [10]. All researchers extracting data from RCT protocols signed confidentiality agreements to conduct the outlined projects according to quality assurance measures and to confidentially handle the information contained in REC files. Only aggregated data will be published, and none of the primary studies, investigators, or sponsors will be identifiable. The final database will only contain data with coded trial identification numbers.

### Data collection for the ASPIRE study

We will use a web-based, password-protected data extraction tool (http://www.squiekero.org) for data collection and data storage [10]. The data extraction sheets in the form of electronic database entry forms were developed and piloted by our team with protocols from the REC in Basel. We compiled a manual with definitions and rules for data extraction for each variable. We will extract the following data for the ASPIRE study:

1. Information on centre and protocol (e.g. sponsor, funding source(s))
2. Trial characteristics (e.g. medical field, type of patient, intervention, number of centres, number of study arms, planned sample size)
3. Specific individual SPIRIT checklist items [12] (whether they are reported in the protocol: “Yes”, “No”, or “Not applicable”)

The complete SPIRIT checklist includes 270 individual components grouped under 33 separate headings. For instance, the heading “sample size calculation” has nine components which are relevant to the calculation of sample size, including but not limited to the statistical test used to calculate the sample size, type I error, type II error, and the minimum anticipated difference or event rate. For our assessment, we will consider all 33 major items or subitems indicated by letters (e.g. 18a, 18b) of the SPIRIT checklist. However, we need to operationalize the checklist for data extraction purposes, i.e. some of the individual components of SPIRIT items or subitems will not be considered, because we feel that such a level of detail is not helpful for our empirical analysis and adds unnecessary complexity and burden for data extractors. The process of identifying which components to include in data extractions was as follows. First, all items and components were included where the heading related to the formulation of a research question using the PICO structure (Population, Intervention, Comparator and Outcome). These were headings that defined the target population, defined the intervention used and any comparators, and defined the outcomes of the study. These headings were considered important because they are relevant to defining the research question of interest for each clinical trial. Likewise, all SPIRIT items were included for headings related to sample size calculation, random sequence generation, allocation concealment, and blinding. These headings were chosen because of their importance for reducing bias in clinical trials.

For the remaining SPIRIT headings, two investigators (AO and BN) independently reviewed each of the items and components under the heading and selected components that encompassed the core message of the heading. These selections were compared and reviewed with three additional collaborators (SH, MB, and Prof. Douglas G. Altman) to achieve consensus on the final selection. A copy of the data extraction forms is provided in Additional file 1, and a list of the 64 items and components selected from the SPIRIT checklist for assessment is provided in Additional file 2.

We will extract data from included RCT protocols in teams of two researchers with methodological training working independently with subsequent agreement checks and consensus discussions in case of discrepancies. Each reviewer will be trained and successfully complete calibration exercises before starting data extraction. Our aim is to extract more than 80% of all included RCT protocols independently and in duplicate; single extractions will only be performed by experienced data extractors (having extracted at least 100 RCT protocols in duplicate before doing single extractions) to minimize extraction errors.

### Data collection for Subproject 1 (DISCO II study)

We will review all eligible RCT protocols and other available REC files for details regarding registration numbers for any primary trial registry. We will search the WHO International Clinical Trials Registry Platform (www.who.int/ictrp), clinicaltrials.gov, and the European Clinical Trials Database (EudraCT) for a corresponding registered RCT, and for those without an obvious registration number, we will consider the population, intervention, control, or primary outcome of a respective RCT as search terms in combination with the name of the principal investigator. For RCTs where we are unable to identify a corresponding record in any of these registries, we will use the Google Web search engine. If a registration number cannot be found, we will categorize RCT protocols as “not registered”. Of the registered protocols, we will extract the date of first registration and the date of entry of the first RCT participant, sponsor, funding source(s), planned sample size, recruitment milestones, primary outcome(s), date of last update, status of the trial, and if results are available in each registry. We define “prospectively registered protocols” as protocols with a date of first registration within a month of the entry date of the first RCT participant to allow for processing delays in the registry and to ensure comparability with previous studies examining trial registration (e.g. [41, 42]).

We will evaluate how many approved RCT protocols result in a peer reviewed full-text publication, how many provide results in a trial registry, and how many have both, peer-reviewed publications and results published in a trial registry. Full texts will be identified directly from trial registries or through individual searches of PubMed and Scopus (one of the most comprehensive databases [43]), in addition to Google Scholar and the Google Web search engine if necessary [44]. We will contact the principal investigator of an RCT to inquire whether there is a corresponding publication in the following cases: (i) no registry entry and no publication can be identified or (ii) the RCT is registered and the study completion date was more than 2 years prior and no full-text publication was identified and results are not published in the trial registry. In case that the study is registered and the status is “ongoing”, we will contact the principal investigator to ask if the status of the clinical trial registry is correct or if the RCT was completed or discontinued. We will extract in duplicate the same information items from full-text publications as from trial registries (see above).

We plan to contact investigators of RCTs, which (i) were neither registered nor published, (ii) were registered and labelled as “ongoing” but the status has not been updated within the last 2 years, or (iii) for which any of the following outcomes remains unclear: prospective registration, premature discontinuation, reason for discontinuation, or non-publication.

### Data collection for Subproject 2 (Patient-reported outcomes study)

In RCT protocols with specified patient-reported outcomes, we will extract (i) whether they specifically consider HRQoL outcomes, (ii) all specified individual patient-reported outcomes, (iii) whether any patient-reported outcome is specified as a primary outcome, (iv) the type of instrument used, (v) whether there is any evidence of validation of the instrument provided, (vi) whether there is an explicit hypothesis specified, (vii) the method of data collection for each patient-reported outcome, (viii) whether a patient-reported outcome is considered in the power/sample size calculation, and (ix) whether a validated minimal important difference is reported. We based our data extraction form for patient-reported outcomes on the CONSORT PRO (Consolidated Standards of Reporting Trials with patient-reported outcomes) tool [45], developed to improve the reporting of patient-reported outcomes in RCTs, as well as the Food and Drug Administration’s “Guidance for Industry” on the use of patient-reported outcomes in medical product development [30].

We will categorize patient-reported outcomes as assessing patients’ well-being measured by (i) a disease-specific HRQoL measure (e.g. Asthma QoL questionnaire); (ii) a multi- or uni-dimensional generic HRQoL instrument (e.g. Short Form-36); (iii) an overall sense of well-being in one question (holistic HRQoL), (iv) patient-reported physical functioning; (v) patient-reported mental/emotional functioning; (vi) patient-reported social functioning; and (vii) reporting of symptoms (e.g. pain). The definition of HRQoL includes only the first three categories (i–iii).

### Data collection for Subproject 3 (Non-regulated interventions study)

No additional data collection is necessary for this substudy.

### Data collection for Subproject 4 (Subgroups study)

In addition to the data extraction for ASPIRE, we will extract information on whether any subgroup analysis is mentioned in the protocol and, if so, whether the analysis is deemed exploratory or confirmatory, whether a clear hypothesis for a subgroup effect is prespecified, whether a direction for this effect is indicated, and whether interaction testing for this part is predetermined. Also, we will record information on the subgroup variables and outcomes for the subgroup analysis, number of subgroup analyses planned, and whether the subgroup analyses are considered in the sample size calculation (if explicitly planned as confirmatory analysis).

### Data collection for Subproject 5 (Routinely collected data study)

We will extract whether routinely collected data were used in any way to support the planning or conduct of all included RCTs, and document the specific type of routinely collected data (e.g. electronic health records, claims data) and their purpose (e.g. outcome data collection) in respective RCTs.

#### Operationalization of the SPIRIT checklist and statistical analysis of ASPIRE

Data cleaning and analysis will be performed using R version 3.6.1 [46].

The SPIRIT checklist contains 33 different major items. Taking the multiple components and subitems (e.g. #5a-d) into account, we prespecified a total of 64 variables to be extracted from each trial protocol to measure adherence to SPIRIT. These 64 variables can take the values “Yes”, “No”, or “Not applicable”. Different scenarios in terms of data structure are possible depending on the complexity of each SPIRIT item:

##### Single SPIRIT items (type 1 variables)

SPIRIT items only requiring a single variable (*n* = 19 items and *n* = 19 variables in total; SPIRIT items number 1–4, 7–9, 13, 19, 22–25, 27–30, 32–33).

##### Multiple component items (type 2 variables)

SPIRIT items requiring more than one variable (*n* = 4 SPIRIT items: 10 (3 variables), 12 (3 variables), 14 (6 variables), 15 (3 variables)).

##### Multiple explicit subitems (type 3 variables)

SPIRIT items which consist of multiple subitems and for which we extracted one variable for each subitem (*n* = 8 SPIRIT items: 5 (a–d), 6 (a, b), 16 (a–c), 18 (a, b), 20 (a–c), 21 (a, b), 26 (a, b), 31 (a–c)).

##### Multiple explicit subitems with several components in subitems (type 4 variables)

SPIRIT items which consist of multiple subitems and for which we extracted more than one variable for one of the subitems (*n* = 2 SPIRIT items: 11 (a–d) with 2 variables for 11a, and 17 (a, b) with 3 variables for 17a).

We will use three different approaches to calculate adherence to the SPIRIT checklist (Table 1).

**Table 1:** Illustration of assigning points for adherence to SPIRIT checklist items according to different approaches

SPIRIT item number	Item hierarchy	Value	Assigned points according to approach		
			Major items (simple)	Major items (allowing for partial credit)	All items
1 (total)	Major item	Yes	1	1	1
…	…	…	…	…	…
17 (total)	Major item	No	0	0.8334	3
17a.1	Component 1 of subitem 17a	No	n/a	0.1667	1
17a.2	Component 2 of subitem 17a	Yes	n/a	0.1667	1
17a.3	Component 3 of subitem 17a	No	n/a	0	0
17b	Subitem 17b	Yes	n/a	0.5	1
…	…	…	…	…	…
20 (total)	Major item	Yes	1	1	3
20a	Subitem 20a	Yes	n/a	0.3333	1
20b	Subitem 20b	Yes	n/a	0.3333	1
20c	Subitem 20c	Yes	n/a	0.3333	1
…	…	…	…	…	…

*n/a* not applicable, *SPIRIT* Standard Protocol Items: Recommendations for Interventional Trials

In the primary analysis, we will use the following approach:

1. *Major item approach (allowing for partial credit of individually met subitems or components of major SPIRIT items):* for each type 1 variable, we will assign one point for each “Yes” or “Not applicable”. We will assign a fraction of one point for each sub-variable in type 2 and type 3 variables. For example, if there are two sub-variables, each will be assigned 0.5 points for a “yes”. In case there are 3 sub-variables, each will be assigned 1/3 point. For type 4 variables, we will apply the same rule, i.e. for example item #17 consists of 17a and 17b. Each of these will be assigned 0.5 points in case of a “yes” or “not applicable”; however, #17a consists of three components, and therefore, each of these type 4 variables will be assigned 1/3 of 0.5 (=0.1667) points in case of a “Yes” or “Not applicable”. A “No” will lead to zero points in each case. The maximum possible score with this approach will be 33 points.

In sensitivity analyses, we will use the following approaches:

2. *Major item approach (simple):* For each of the 33 major SPIRIT items, we will assign one point for each “Yes” or “Not applicable” in type 1 variables, and one point if all type 2, type 3, and type 4 variables contingent to a major SPIRIT item are “Yes” or “Not applicable”. Otherwise, zero points will be assigned. The maximum possible score with this approach will be 33 points.
3. *All item approach:* For each “Yes” or “Not applicable” in each variable (types 1, 2, 3, or 4), we will assign one point. A “No” will be assigned zero points. The maximum possible score with this approach will be 64 points.

Regarding major item 17 of SPIRIT in Table 1, for instance, component 1 of subitem 17a (blinding status of participants) is reported, component 2 of subitem 17a (blinding status of care providers) is reported, component 3 of subitem 17a (blinding status of outcome assessors) is not reported, and subitem 17b (conditions when unblinding is permissible) is reported. With the “simple Major item approach”, we assign item 17 a total of 0, because not all components of subitems were reported; with the “Major item approach allowing for partial credit”, a total of 0.8334 is assigned, because only one component of subitem 17a was not reported; and with the “all item approach”, we assign a total of 3 points, because all reported components or subitems receive a point.

In further sensitivity analyses, we will repeat the calculations with each of the mentioned approaches but will assign points only in case of a “Yes” for a specific variable; in case of “Not applicable”, we will assign neither one nor zero points, but will not consider this item for a specific protocol. This means that the maximum possible score could vary across protocols for each of the three approaches.

Our main outcome will be adherence to SPIRIT checklist items reported in RCT protocols approved by RECs in 2012 and in 2016. We will calculate adherence as the proportion of trial protocols that address each individual SPIRIT checklist item (according to the different approaches described above) as the mean/median number of items adhered to per protocol. Our main analyses will be based on the major item approach allowing for partial credit of individually met subitems or components of major SPIRIT items with “not applicable” getting assigned a point because it keeps the hierarchical structure of the SPIRIT checklist and it independently considers all components and subitems of all individual SPIRIT items.

For descriptive analyses, we will stratify included protocols by the year of approval (2012 versus 2016), sponsorship (industry versus investigator), sample size (above vs below/equal to the median sample size), centre status (single vs multicentre RCTs), and reported methodological support (yes vs no). To analyse whether the following independent variables are associated with adherence to a larger proportion of SPIRIT items (dependent variable), we will use multivariable regression models (beta regression [47]—primary analysis, and hierarchical logistic regression):

1. Year 2012 versus 2016 (*Hypothesis:* RCT protocols approved in 2016 are more comprehensive due to SPIRIT)
2. Investigator sponsorship versus industry sponsorship (*Hypothesis:* industry-sponsored RCT protocols are more complete and better structured, thus associated with a larger proportion of adherence to SPIRIT items)
3. Sample size (in 1000 increments) (*Hypothesis:* larger trials are better planned and have more comprehensive protocols, thus associated with a larger proportion of adherence to SPIRIT items)
4. Single-centre versus multicentre RCTs (*Hypothesis:* multi-centre RCTs are better planned and have more comprehensive protocols, thus associated with a larger proportion of adherence to SPIRIT items)
5. Lack of methodological support versus support from a Clinical Research Organization or Clinical Trial Unit (*Hypothesis:* protocols mentioning methodological support are more comprehensive, thus associated with a larger proportion of adherence to SPIRIT items)

To directly model the proportion of the SPIRIT items adhered to per protocol, we will use beta regression analysis [47]. Using the aggregated proportion as a response does not allow us to capture the variability within each protocol. Therefore, we will additionally consider a hierarchical logistic regression model with two levels: the “SPIRIT item level” and the “protocol level”. The response is a binary variable indicating adherence to each SPIRIT item with clustering by protocol. In this approach, we will include the covariables of interest as fixed effects and the protocol as a random effect.

For all types of regression analyses, we will report coefficients or odds ratios (ORs) accompanied by 95% confidence intervals (CIs). To specifically test our hypotheses that investigator-sponsored protocols improved in terms of adherence to SPIRIT between 2012 and 2016 while industry-sponsored protocols did not (potentially due to a ceiling effect), we will include a corresponding interaction term (year of approval * sponsorship) in each of the mentioned multivariable regression models. We will use the same approach to test our hypothesis that methodologically supported protocols (involvement of the Clinical Trial Unit or Clinical Research Organization) improved less than RCT protocols without reported methodological support.

We will provide descriptive statistics as frequencies and proportions for binary data and mean/median, minimum, maximum, and standard deviation/interquartile range (IQR) for continuous data. All statistical tests will be performed at a significance level of 0.05 unless specified otherwise.

#### Statistical analyses for substudies

##### Subproject 1 (DISCO II study)

We will assess how many RCTs are (i) registered, (ii) published (results in peer-reviewed journal or trial registry), (iii) registered and published (peer reviewed journal or trial registry), or (iv) neither registered nor published. In addition, we will assess how many registered RCTs were registered prospectively (within 1 month of recruiting first patient) and how many post hoc, and we will assess the proportion of unpublished RCTs that were registered (prospectively or post hoc).

We will descriptively analyse RCTs that were prematurely discontinued and list the frequencies and proportions for specific reasons for discontinuation, stratified by industry and investigator sponsorship as we have previously done [10] to allow for comparison before and after publication of the SPIRIT guideline (2000–2003 vs 2012 vs 2016). We will further stratify our analyses by country of RECs and descriptively compare proportions to see whether there is any evidence for heterogeneity across countries. We will use data from the main ASPIRE study together with DISCO II study data to conduct two multivariable regression analyses. In the first regression, “trial discontinuation due to poor recruitment” will be the dependent variable and the proportion of reported SPIRIT items will be the main independent variable of interest to see whether we can find evidence for an association between protocol comprehensiveness and the risk for trial discontinuation due to poor recruitment. Our hypothesis is that less comprehensive protocols are correlated with poorly planned trials leading to a higher risk of recruitment failure and trial discontinuation. In a second multivariable regression, “trial non-publication” will be the dependent variable and, again, the proportion of reported SPIRIT items will be the main independent variable to explore for an association between protocol comprehensiveness and non-publication of trials. Our hypothesis is that less comprehensive protocols are correlated with less professional trial conduct and result in reporting leading to a higher risk of non-publication.

In an additional study, we will assess if specific RCT characteristics are different between approved protocols, information in trial registries, and publications. Specifically, we will check for differences in the following characteristics:

- Primary outcome
- Planned sample size
- Blinding of patients, care givers, or outcome assessors
- Funding
- Patient-reported outcomes
- Planned subgroup analyses
- RCT results (published in peer reviewed journals vs trial registries)

##### Subproject 2 (Patient-reported outcomes study)

Descriptive analysis of RCT characteristics and patient-reported outcome data.

##### Subproject 3 (Non-regulated interventions study)

Descriptive analysis of ASPIRE data stratified by RCTs with “regulated interventions” such as drugs, biologics, or medical devices, and “non-regulated interventions” such as dietary interventions, surgical procedures, behavioural and lifestyle interventions or exercise programmes, and others. We will use the same multivariable regression models as described for the ASPIRE study but include “regulated intervention” (yes vs no) as an additional independent variable. To test for an effect modification with year of REC approval (2012 vs 2016), we will add a corresponding interaction term (year of approval * regulated intervention) to the regression model. Our hypothesis is that RCT protocols with regulated interventions are better planned due to the more stringent regulatory requirements and, therefore, have more comprehensive protocols associated with a larger proportion of adherence overall; however, the improvement in adherence of RCT protocols from 2012 to 2016 may be more pronounced in RCT protocols with non-regulated interventions.

##### Subproject 4 (Subgroups study)

Descriptive analysis of RCT characteristics with respect to planned subgroup analyses.

##### Subproject 5 (Routinely collected data study)

Descriptive analysis of RCT characteristics and types and purposes of routinely collected data to support RCTs.

## Discussions

The ASPIRE study and the five outlined substudies have the overall aim to monitor and ultimately inform improvements in the planning, conduct, analysis, and reporting of RCTs. Our findings will inform multiple aspects of RCT design, protocol completeness, and practical aspects such as trial registration, premature discontinuation, and non-publication of RCTs observing potential changes over time. There may be further studies making use of the collected data, in particular potential comparisons of RCT information documented in trial registries with trial protocols and with corresponding publications.

### Strengths and limitations

Strengths of our proposed studies include full access to protocols and associated documents of all included RCTs approved by RECs in Switzerland, the UK, Germany, and Canada in 2012 and 2016. Involved researchers are formally trained methodologists, and we will use standardized methods of data collection. We will pre-pilot all data extraction forms with detailed instructions and carry out calibration exercises to align study processes. With all Swiss RECs participating in this international study, the data will be highly representative of Switzerland and will allow us to explore for differences between Swiss and other RECs. We specifically planned to conduct a subgroup analysis to investigate whether the completeness of Swiss RCT protocols is different from non-Swiss RCT protocols, because a new federal Law on Research in Humans (Human Research Act) and its subsidiary ordinances came into effect in January 2014. Consequently, the roles and operating procedures of the Swiss RECs and the drug licencing authority Swissmedic were revised. In this context, new guidance documents for trial protocols that built on the SPIRIT recommendations (www.swissethics.ch) were promoted to Swiss researchers.

Our study has several limitations. First, we are using a convenience sample of 21 RECs outside of Switzerland (Freiburg in Germany, Hamilton in Canada, and 19 RECs of the Bristol regional office). We cannot say whether they are representative of other RECs in these or other countries; however, to our knowledge, they are not in any way particular. Second, since we will include RCT protocols in the ASPIRE study that have already been approved by RECs, SPIRIT items such as “research ethics approval” and “consent forms provided” will always be fulfilled and will, therefore, not contribute to discriminate more complete protocols from less complete protocols. Third, in our operationalization of the SPIRIT checklist for data extraction purposes, we did not consider all individual components of each SPIRIT item but included all major items and selected components as described in our methods section. Involved experts felt that not all 270 individual components of SPIRIT items were necessary for the study and considering all would add unnecessary complexity and burden for data extractors. Finally, our assumption that the adherence to SPIRIT as a measure for the completeness of reporting of RCT protocols indeed reflects the “quality of RCT conduct in general” is based on scientific reasoning and common sense rather than empirical evidence. To address this question, we designed the DISCO II substudy to further investigate the association of protocol adherence to SPIRIT and premature discontinuation due to poor recruitment or non-publication of RCTs.

### Significance

The impact of poorly planned RCTs is pervasive to the entire research process, wastes scarce resources, and may have harmful consequences for all stakeholders including patients, decision makers, and the scientific community, thus affecting society as a whole. To better understand and ultimately improve the clinical research process, and RCTs in particular, it is necessary to empirically and systematically investigate the design, methods, and dissemination of recent RCTs. The present international study of RCT protocols approved in 2012 or 2016 will provide information on the completeness of trial protocols and potential changes between 2012 and 2016. Our plan of research will identify reporting deficiencies and associated RCT characteristics and clarify whether protocol adherence to SPIRIT recommendations is associated with the proportion of prematurely discontinued RCTs or the proportion of unpublished RCTs. Our study will investigate the use of patient-reported outcomes and HRQoL outcomes in RCTs over time; compare characteristics of RCTs testing regulated interventions versus non-regulated interventions; examine the planning of subgroup analyses in RCTs over time; and assess the use and specific purposes of routinely collected data to support RCTs.

## Supplementary information

**Additional file 1.** Data extraction form for the Adherence to SPIrit REcommendations (ASPIRE) Study.

**Additional file 2.** List of the 64 SPIRIT checklist items and components for the Adherence to SPIrit REcommendations (ASPIRE) Study.

## Data Availability

The data supporting the conclusions of this article is included within the article (and its additional file).

## Abbreviations

ASPIRE: Adherence to SPIrit REcommendations
CI: Confidence interval
CONSORT PRO: Consolidated Standards of Reporting Trials with Patient-Reported Outcomes
DISCO study: DISCOntinued trials study
EHR: Electronic health record
EudraCT: European Clinical Trials Database
HRQoL: Health-related quality of life
HTA: Health Technology Assessment
ICMJE: International Committee of Medical Journal Editors
IQR: Interquartile range
NIHR: National Institute for Health Research
OR: Odds ratio
QoL: Quality of life
RCT: Randomized clinical trial
REC: Research ethics committee
SPIRIT: Standard Protocol Items: Recommendations for Interventional Trials
UK: United Kingdom
