# Supplementary material for: Rationale and design of repeated cross-sectional studies to evaluate the reporting quality of trial protocols: the Adherence to SPIrit REcommendations (ASPIRE) study and associated projects
Source: Trials. 2020 Oct 28;21:896. doi: 10.1186/s13063-020-04808-y (PMC7594472; doi:10.1186/s13063-020-04808-y)
Supplement: Supplementary file 1 — Additional file 1. Data extraction form for the Adherence to SPIrit REcommendations (ASPIRE) Study. [file 13063_2020_4808_MOESM1_ESM.pdf]

Appendix A

Data extraction form for the Adherence to SPIrit REcommendations (ASPIRE) Study

Label	Options
1. Country of Ethics Committee	
2. Name of Ethics Centre	
3. Local Ethics Identification Number	
4. Sponsor name (title, first name, surname, company if applicable)	
5. Sponsor email address	
6. Site/Location of overall study initiation (PI affiliation)	Switzerland
	Other
	Not reported
If site initiation in Switzerland, please provide name and location of institution:	
7. Study Acronym	
8. Study Title (Exact Quote)	
9. Date of Ethics Application	
9a. Date of first RESPONSE by Ethics Committee (does not need to be the same as approval date)	
9b. Response category (Switzerland specific, others select "not applicable")	A positiv
	B positiv mit Bemerkung
	C mit Auflage, Nachbegutachtung notwendig
	C mit Auflage, schriftliche Mitteilung ausreichend
	D negativ
	E Nicht-Eintreten
	Not applicable as Ethics Committee not in Switzerland
10. Date of first APPROVAL by Ethics Committee	
11. Clinical Area	Medical
	Surgical
	Paediatrics
	Other
If medical area, choose from list	Neurology
	Cardiovascular
	Respiratory
	Gastro/intestinal
	Nephrology
	Rheumatology
	Infectious Disease

	Oncology
	Intensive Care
	Hematology
	Endocrinology
	Dermatology
	Anaesthetics
	Psychiatry
	Other
If surgical area, choose from list	General Surgery
	Obstetrics/Gynecology
	Neurosurgery
	Ophthalmology
	Ear-nose-throat (ENT)
	Cardiothoracic
	Urology
	Orthopedics
	Plastic Surgery
	Other
If pediatrics area, choose from list	Neurology
	Cardiovascular
	Respiratory
	Gastro/intestinal
	Nephrology
	Rheumatology
	Infectious diseases
	Oncology
	Intensive care
	Hematology
	Endocrinology
	Dermatology
	Anaesthetics
	General surgery
	Neurosurgery
	Ophthalmology
	Ear-nose-throat (ENT)
	Cardiothoracic
	Urology
	Orthopedics
	Plastic Surgery
	Other
12. Trial Registration Number	
13. Trial Registry Name	Clinicaltrials.gov
	ISRCTN

	EudraCT
	ANZCTR
	Not reported
	Other (please specify)
14. Swiss Human Research Act Risk Category	A
	B
	C
	Not applicable
	Not reported
15. Is trial labelled as pilot or feasibility trial?	Yes
	No
16. Is it a dose finding trial?	Yes
	No
17. Hypothesis (check all that apply)	Superiority
	Non-inferiority / Equivalence
	Not labelled in this regard / unclear
18. Please copy the primary outcome(s) from the protocol	
19. Are any outcomes specifically labelled as “adverse events”, “adverse effects”, “side effects”, or "tolerability" ?	Yes
	No
If yes, adverse events (or synonyms thereof) are...	not further specified (e.g. the term adverse events is just mentioned under outcome section)
	specifically defined (e.g. specific types of adverse events such as rash, itching, nausea etc. are mentioned)
20. Is a patient-reported outcome specified (an outcome that comprises information reported by a patient or a caregiver (parent or guardian))?	Yes
	No
If yes: the specified patient-reported outcome captures the following information (check all that apply):	Symptoms (pain, headaches, sleeplessness, etc.)
	Physical functioning
	Mental/emotional functioning
	Social functioning
	Disease-specific outcome measure (eg. Asthma QoL questionnaire, Beck Depression Inventory)

	Multidimensional health-related quality of life (HRQL; eg. SF-36)
	Overall sense of well-being in one question (holistic HRQL; eg. captured with a VAS)
	Satisfaction with treatment
	Utility (an individual's preferences/values for certain health states/outcomes)
	Other (please specify)
If yes: patient-reported outcome + measurement instrument	
If yes, patient-reported outcome used for sample size calculation?	Yes
	No
If yes, minimal important difference (MID) mentioned?	Yes
	No
If yes, reference for MID? (please enter full citation or if not reported, enter "NR")	
20a. Is routinely collected data used in the study?	Yes
	No
20b. If yes, routinely collected data is used:	For patient identification and/or recruitment?
	As part of the randomized intervention?
	For any of the planned outcomes?
	Other
21. Any planned collection of costs or cost-effectiveness analysis mentioned?	Yes
	No
22. The setting for the majority of recruited patients is (check all that apply)	Community
	Outpatient clinic
	Emergency department
	In-patients hospital care
	Intensive care unit
	Unclear
23. The age-group of patient population is (check all that apply)	Adults (>=16 years)
	Only elderly (>=60)
	Pediatric (<18)
24. Please specify the study population	
25. Estimated sample size/number of participants	
26. Number of overall study centres	
27. If multicentre, national or multinational	National

	International
	Not applicable
28. Number of study centres recruiting in Switzerland (or Canada/Germany if applicable)	
29. Trial Design (check all that apply)	Parallel
	Crossover
	Cluster
	Factorial
	Split Body
	Other
	Not applicable
30. Number of trial arms	
31. Presence of logistic/ methodological support/experience? (check all that apply)	Clinical trial unit (CTU)
	Contract Research Organization (CRO)
	Evidence for ample expertise of the PI/Institution
	Not reported
	Other
32. Please specify the intervention(s)	
33. Intervention category/ies	Drug
	Surgery / Invasive Procedure
	Device
	Vaccine
	Radiation
	Rehabilitation
	Behavioural / Lifestyle / Education / Counselling
	Dietary Supplement
	Other
34. Please specify the control(s)	
35. Type of control(s)	No treatment / Standard care
	Active (drug/other treatment)
	Placebo / Sham
36. Name of funder(s)	
37. Initiation/Sponsorship	Definitely industry initiated
	Probably industry initiated
	Probably investigator initiated

	Definitely investigator initiated
38. Title: Basic study design, patient population, and intervention provided in study title (if applicable trial acronym)? (reporting)	Yes<br>No
39. Trial Registration: Registry name and trial identifier provided? (reporting)	Yes<br>No
40. Protocol: Version Number and date provided? (reporting)	Yes<br>No
41. Funding: Sources of financial and non-financial support declared? (reporting)	Yes<br>No
42. Roles and Responsibilities: Names of protocol contributors/ authors provided? (reporting)	Yes<br>No
43. Roles and Responsibilities: Name and contact details of sponsor provided? (reporting)	Yes<br>No
44. Roles and Responsibilities: Role of sponsor and funder in trial described? (reporting)	Yes<br>No
45. Roles and Responsibilities: Steering Committee General Membership and Role described? (reporting)	Yes<br>No<br>Not applicable
46. Background and rationale: Is research question described and justified? (as a minimum, we expect a systematic search, see info) (reporting)	Yes<br>No
46a. Systematic review on PICO explicitly mentioned in background/introduction?	Yes<br>No
47. Background and rationale: Comparator choice explained? (reporting)	Yes<br>No
48. Objectives: Specific objectives described for each comparison (if multiple)? (reporting)	Yes<br>No
49. Trial design: Trial design described? (trial type (eg, parallel group, crossover, factorial, single group), allocation ratio, and framework (eg, superiority, equivalence, noninferiority, exploratory)) (reporting)	Yes<br>No
50. Study Setting: Are countries where data will be collected listed? (reporting)	Yes<br>No
51. Eligibility criteria: Inclusion and exclusion criteria for trial participants described? (reporting)	Yes<br>No
52. Eligibility criteria: Inclusion and exclusion criteria for study centres and individuals who will perform the intervention described? (reporting)	Yes<br>No<br>Not applicable
53. Intervention(drug): Generic Name, Dose and Schedule of intervention described? (reporting)	Yes<br>No<br>Not applicable
54. Intervention(non-drug): Setting of intervention administration described? (reporting)	Yes<br>No<br>Not applicable

55. Intervention(non-drug): Individuals administering interventions (e.g. expertise) mentioned? (reporting)	Yes
	No
	Not applicable
56. Interventions - Modifications: Standard criteria for modifications of interventions described? (reporting)	Yes
	No
	Not applicable
57. Interventions - Adherence: Are strategies to improve adherence or any procedures for monitoring adherence described? (reporting)	Yes
	No
	Not applicable
58. Interventions - Concomitant care: Permitted care and interventions during trial described? (reporting)	Yes
	No
59. Primary Outcome: Specific measurement variable described? (reporting)	Yes
	No
	Not applicable
60. Primary Outcome: Analysis metric (e.g. change from baseline) described? (reporting)	Yes
	No
	Not applicable
61. Primary Outcomes: Is time point of measurement mentioned? (reporting)	Yes
	No
	Not applicable
62. Participant timeline: Timing of visit for participants described (e.g. schematic diagram)? (reporting)	Yes
	No
63. Sample size: Estimated number total or per group mentioned? (reporting)	Yes
	No
64. Sample size: Outcome used for samples size calculation described? (reporting)	Yes
	No
	Not applicable
65. Sample size: Assumed values for outcome in each study group provided? (reporting)	Yes
	No
	Not applicable
66. Sample size: Rationale or reference for assumed outcome values provided? (reporting)	Yes
	No
	Not applicable
67. Sample size: Type of statistical test provided? (reporting)	Yes
	No
	Not applicable
68. Sample size: Alpha value provided? (reporting)	Yes
	No
	Not applicable
69. Sample size: Statistical Power provided? (reporting)	Yes
	No
	Not applicable
	Yes

70. Sample size: Adjustment for missing data, if relevant, described? (reporting)	No
	Not applicable
71. Sample size: Rationale for intended sample size if not derived statistically provided? (reporting)	Yes
	No
	Not applicable
72. Recruitment: Location of participant recruitment described? (reporting)	Yes
	No
73. Recruitment: Person(s) who will recruit participants decribed? (reporting)	Yes
	No
74. Recruitment: Expected recruitment rate provided? (reporting)	Yes
	No
75. Recruitment: Estimated number or rate of eligible patients	
76. Recruitment: Estimated duration of the patient recruitment	
77. Recruitment: Monitoring of recruitment during trial mentioned? (reporting)	Yes
	No
78. Recruitment: Financial and non-financial incentives for participants described? (reporting)	Yes
	No
	Not applicable
79. Recruitment: Financial and non-financial incentives for investigators described? (reporting)	Yes
	No
80. Allocation: Method for generation of random sequence described? (e.g. computer-generated random numbers) (reporting)	Yes
	No
	Not applicable
81. Allocation: Ratio provided? (e.g. 1:1, 2:1) (reporting)	Yes
	No
	Not applicable
82. Allocation: Type of randomization described? (e.g. "simple", block, matched pair, etc.) (reporting)	Yes
	No
	Not applicable
83. Allocation: Non-random allocation-method described? (reporting)	Yes
	No
	Not applicable
84. Allocation: Rationale for non-random allocation provided? (reporting)	Yes
	No
	Not applicable
85. Allocation: Allocation concealment mechanism described? (reporting)	Yes
	No
	Not applicable
86. Allocation: Person who will enrol/assign participants described? (reporting)	Yes
	No
	Not applicable
87. Blinding: Status of participants described? (reporting)	Yes
	No

88. Blinding: Status of care providers described? (reporting)	Yes<br>No
89. Blinding: Status of outcome assessors described? (reporting)	Yes<br>No
90. Blinding: Conditions when unblinding is permissible mentioned? (reporting)	Yes<br>No<br>Not applicable
91. Data Collection: Personnel who will collect data specified? (reporting)	Yes<br>No
92. Data collection: Strategies to promote participant retention and complete follow-up described? (reporting)	Yes<br>No
93. Data Management: Data entry and coding processes described? (reporting)	Yes<br>No
94. Statistical Methods: Main analysis for primary outcome including analysis methods for statistical comparisons described? (reporting)	Yes<br>No
95. Statistical Methods: Handling of missing data defined? (reporting)	Yes<br>No<br>Not applicable
96. Statistical Methods: Effect measure for primary analysis clearly specified? (e.g. risk ratio, odds ratio etc.) (reporting)	Yes<br>No
97. Statistical Methods: Significance level specified? (e.g. alpha of 5% or p<0.05) (reporting)	Yes<br>No
98. Statistical Methods: Use of confidence intervals mentioned? (e.g. "results will be accompanied by a confidence interval") (reporting)	Yes<br>No
99. Statistical Methods: Definition of subgroup categories provided? (reporting)	Yes<br>No<br>Not applicable
100. Any subgroup analysis mentioned (this question triggers a set of questions for a subproject independent of SPIRIT)?	Yes<br>No
If yes, is it explicitly mentioned that subgroup analyses are exploratory?	Yes<br>No
If yes, is a clear hypothesis for a subgroup effect pre-specified?	Yes<br>No
If yes, is a clear hypothesis with a direction of subgroup effect pre-specified?	Yes<br>No
If yes, use of interaction test for subgroup analysis mentioned?	Yes<br>No
If yes, please list planned subgroup variables	
If yes, please list planned outcomes for subgroup analyses	
If yes, please specify number of subgroup analyses planned (=SG variables x outcomes)	
If yes, subgroup analysis considered in sample size calculation?	Yes<br>No

101. Statistical Methods: Does the protocol define which participants will be included in the main analysis in terms of protocol adherence and missing data? (reporting)	Yes
	No
102. Data Monitoring Committee: Is a data monitoring committee planned for this study?	Yes
	No
103. Data Monitoring Committee: Is it explicitly reported whether a DMC is planned or why it is not planned? (reporting)	Yes
	No
104. Data Monitoring: Planned number of interim analyses	
105. Data Monitoring: Purpose of interim analyses (check all that apply)	Benefit
	Harm
	Futility
	Sample size recalculation
	No reason provided
	Not applicable
	Other
106. Data Monitoring: Reported who has ultimate authority to stop the trial? (reporting)	Yes
	No
107. Data Monitoring: Does the sponsor retain the right to stop the trial?	Yes
	No
	Not reported
If yes, explicitly at any time for any reason?	Yes
	No
108. Harms: Plans for collecting, assessing, reporting, managing anticipated/unanticipated adverse events provided? (reporting)	Yes
	No
109. Auditing: Procedures of audits and/or external monitoring described (e.g. clinical trial unit/CROs)? (reporting)	Yes
	No
	Not applicable
110. Research Ethics Approval: Where approval has been obtained, or plans for seeking approval, provided? (should always be yes in this study) (reporting)	Yes
	No
111. Protocol Amendments: Process for making amendments described? (reporting)	Yes
	No
112. Consent or Assent: Informed Consent process described? (reporting)	Yes
	No
113. Consent or Assent – Ancillary Studies: Process to obtain additional consent for collection and use of data and biological specimens described? (reporting)	Yes
	No
	Not applicable
114. Confidentiality: Described how data will be collected, kept secure, and maintained during the trial? (reporting)	Yes
	No
115. Declaration of Interests: Financial and other competing interests clearly stated? (reporting)	Yes
	No
116. Access to data: Is it clearly mentioned who will have access to full dataset after trial completion? (reporting)	Yes
	No
	Yes

117. Ancillary and post-trial care: Any plans to provide or pay for ancillary care during the trial provided? (reporting)	No
118. Dissemination Policy: Plans to disseminate trial results to key stakeholders/publication provided? (reporting)	Yes
	No
119. Dissemination Policy: Does the protocol mention any rules/regulations between the investigators and the sponsor with respect to the rights of publication of the trial results? (reporting)	Yes
	No
	Not applicable
If yes, please copy the corresponding statement from the protocol	
If yes, which statement suits best?	Only the sponsor retains the right to analyze and publish the data (no cooperation with investigators at all)
	The sponsor retains the right to approve any manuscript/abstract before publication (sponsor retains explicitly the right to reject submission for publication)
	The sponsor retains at least the right to review and comment on any manuscript/abstract before publication
	Free publication rights for the investigators, no constraints at all by the sponsor (sponsor has explicitly NOT the right to reject the submission for publication)
	Protocol refers to a separate publication agreement between sponsor and investigator
	Other (Please enter description for other)
120. Dissemination Policy: Authorship eligibility criteria described?	Yes
	No
121. Dissemination Policy: Plans for granting access to full trial protocol provided? (reporting)	Yes
	No
122. Informed Consent Materials: Model consent and/or assent forms provided (e.g in Appendix)? (reporting)	Yes
	No
	Yes

123. Biological Specimens: Details of specimen collection provided? (reporting)	No
	Not applicable
124. Any comments?	
