# Supplementary material for: Rationale and design of repeated cross-sectional studies to evaluate the reporting quality of trial protocols: the Adherence to SPIrit REcommendations (ASPIRE) study and associated projects
Source: Trials. 2020 Oct 28;21:896. doi: 10.1186/s13063-020-04808-y (PMC7594472; doi:10.1186/s13063-020-04808-y)
Supplement: Supplementary file 2 — Additional file 2. List of the 64 SPIRIT checklist items and components for the Adherence to SPIrit REcommendations (ASPIRE) Study. [file 13063_2020_4808_MOESM2_ESM.pdf]

Appendix B

List of the 64 SPIRIT checklist items and components for the Adherence to SPIrit REcommendations (ASPIRE) Study

Variable	Spirit Item Number
Title: Basic study design, patient population, and intervention provided in study title	1
Trial Registration: Registry name and trial identifier provided	2
Protocol version, number and date	3
Funding sources of financial and non-financial support declared	4
Names of protocol contributors/ authors	5a
Name and contact details of sponsor	5b
Role of sponsor and funder in trial	5c
Steering committee general membership and role described	5d
Research question described and justified	6a
Comparator choice explained	6b
Specific objectives described	7
Trial design described	8
Countries where data will be collected listed	9
Inclusion and exclusion criteria for trial participants described	10
Inclusion and exclusion criteria for study centres and individuals who will perform the intervention described?	10
Individuals administering interventions (non-drug)	10
Generic name, dose and schedule of intervention	11a
Setting of intervention administration	11a
Criteria for modifications of interventions	11b
Strategies to improve or monitoring of adherence	11c
Permitted concomitant care	11d
Primary Outcome: specific measurement variable	12
Primary outcome: analysis metric	12
Primary outcomes: time point of measurement	12
Participant timeline: Timing of visit for participants described	13
Sample size: or per group	14
Sample size: outcome used for samples size calculation	14
Sample size: assumed values for outcome	14
Sample size: alpha value	14
Sample size: Statistical Power	14
Sample size: rationale for sample size if not derived statistically	14
Location of participant recruitment	15
Person(s) who will recruit participants	15
Expected recruitment rate	15
Method for generation of random sequence	16a
Allocation concealment mechanism	16b

Person who will enroll/assign participants	16c
Blinding status of participants	17a
Blinding status of care providers	17a
Blinding status of outcome assessors	17a
Conditions when unblinding is permissible	17b
Personnel who will collect data	18a
Strategies to promote participant retention and complete follow-up	18b
Data entry and coding	19
Main analysis for primary outcome	20a
Definition of subgroup categories	20b
Definition of analysis population	20c
DMC is planned or why it is not planned	21a
Who has authority to stop the trial	21b
Anticipated/unanticipated adverse events collection	22
Audits/external monitoring described	23
Research ethics approval	24
Process for making amendments described	25
Informed Consent process described	26a
Process to obtain additional consent for collection and use of data and biological specimens	26b
Confidentiality: Described how data will be collected, kept secure, and maintained during the trial	27
Declaration of Interests: financial and other competing interests clearly stated	28
Who will have access to full dataset	29
Ancillary and post-trial care: Any plans to provide or pay for ancillary care during the trial provided	30
Plans to disseminate trial results to key stakeholders/publication provided	31a
Authorship eligibility criteria	31b
Plans for granting access to full trial protocol	31c
Consent forms provided	32
Details of specimen collection	33
